# Preparation and Evaluation of Thermosensitive Liposomes Encapsulating I-125-Labeled Doxorubicin Derivatives for Auger Electron Therapy

**DOI:** 10.3390/molecules28041864

**Published:** 2023-02-16

**Authors:** Mohamed Elsaid Nasr Elghobary, Masayuki Munekane, Kenji Mishiro, Takeshi Fuchigami, Kazuma Ogawa

**Affiliations:** 1 Graduate School of Medical Sciences, Kanazawa University, Kanazawa 920-1192, Japan; 2 Institute for Frontier Science Initiative, Kanazawa University, Kanazawa 920-1192, Japan

**Keywords:** thermosensitive liposomes, I-125-labeled doxorubicin derivatives, Auger electron therapy, hyperthermia

## Abstract

Auger electrons (AEs) are very low-energy electrons emitted by radionuclides such as I-125 (^125^I). This energy is deposited across a small distance (<0.5 μm), resulting in high linear energy transfer that is potent for causing lethal damage to cancer cells. Thus, AE-emitting radiotherapeutic agents have great potential for cancer treatment. In this study, thermosensitive liposomes (TSLs) encapsulating ^125^I-labeled doxorubicin (DOX) derivatives were developed for Auger electron therapy, targeting the DNA of cancer cells. A radioiodinated DOX derivative [^125^I]**5** highly accumulated in the nuclei of cancer cells and showed potent cytotoxicity against Colon 26 cancer cells by AEs. Subsequently, [^125^I]**5** was loaded into the TSLs with high encapsulation efficiency. Potent release of [^125^I]**5** from TSLs was achieved with heating, whereas a decreased release was observed without heating. Furthermore, TSLs encapsulating [^125^I]**5** showed a high uptake in the nuclei at 42 °C for 1 h. We supposed that [^125^I]**5** was released by heating at 42 °C and accumulated in the nuclei in the cells. These results suggest that the combination of TSLs encapsulating [^125^I]**5** and hyperthermia is an effective cancer therapy.

## 1. Introduction

Targeted radionuclide therapy (TRT) is a promising approach to cancer treatment as it allows the specific radiation of tumor cells. TRT is a technique of choice for dealing with diffuse and metastatic cancer [1,2]. The cancer cells are killed by radiation (electrons or α particles) emitted by radionuclides conjugated with biological carriers, such as peptides, monoclonal antibodies, their fragments, and any other small biologically active molecules. TRT is attractive because of its high therapeutic efficiency, and many probes have been developed for TRT [3,4]. Among the kinds of radiation used for TRT, Auger electrons (AEs) are very low-energy electrons emitted by radionuclides that decay by internal conversion or electron capture, occurring in large numbers (4.7–36.9 per disintegration). This power is deposited over a small distance (<0.5 μm). This means AEs have higher linear energy transfer (LET) (4–26 keV/µm) than beta particles, with LET of 0.1–1.0 keV/µm, in which power is deposited over 0.1–10 mm [5]. AE-emitters may permit particular therapies capable of high cytotoxicity against cancer cells, even during micrometastases and for single circulating cancer cells [6]. However, they must be sent to specific targets, primarily the nucleus.

Most AE-emitting molecular radionuclide therapy studies have used indium-111 and iodine-125 [6]. Thus, these radionuclides need to be delivered to the nuclei of cancer cells to be effective, because their radiotoxic properties are only active close to DNA. Several techniques based on nuclear localization sequences (NLSs) for translocating antibodies and somatostatin derivatives labeled with AE-emitting radionuclides have been applied to tumor cell nuclei [7,8]. The concept of using NLS to encourage the translocation of radiolabeled cell-targeting agents from the cytoplasm to the nucleus is attractive. However, the NLS-based strategy allows only a small amount of radiolabeled compounds to be sent to the nuclei of cancer cells. Specific nuclear delivery has been achieved using ^125^I-labeled nucleotides, oligonucleotides, steroid hormones, and growth factors. However, it has been recognized that there is a need to improve their delivery to cancer tissues [9,10].

Liposomes are among the most well-known drug delivery carriers that improve the biodistribution of low molecular weight compounds. In recent years, various types of stimuli-responsive liposomes have been reported [11]. Thermosensitive liposomes (TSLs) are among the most attractive ones [12,13]. TSLs used in combination with local hyperthermia led to a 10- to 50-fold increase in liposome extravasation [14] and improved tumor drug uptake and tumor growth delay in mice approximately by 2- to 9-fold (maximum 17-fold) than either no hyperthermia or non-liposomal drug [15].

Doxil is a typical liposomal formulation that encapsulates doxorubicin (DOX), a widely used anticancer anthracycline antibiotic, that accumulates in the nucleus. ^125^I–labeled anthracycline compounds such as DOX and daunorubicin derivatives have been evaluated as candidate radioligands for Auger electron therapy [16]. Anthracyclines can cross cell membranes because of their amphipathic properties. They translocate into the cytoplasm and even the nucleus because of their DNA-intercalating capabilities. Therefore, it is considered that ^125^I-labeled anthracyclines localize the nuclide close enough to the tumor cell DNA to induce double-strand breaks. In addition, liposomes encapsulating ^125^I-labeled daunorubicin derivatives have been developed and specific delivery of the labeled compounds to tumor tissues has been achieved [17,18]. However, these studies failed to evaluate specific drug release in the tumor tissue. Therefore, ^125^I-labeled anthracycline derivatives need to be released from liposomes because only released anthracyclines could accumulate in the nuclei with high efficiency.

**Figure 1:**
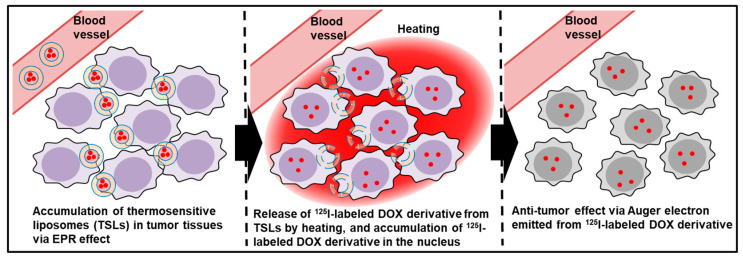
A schematic diagram. Strategy for Auger electron therapy using TSLs and hyperthermia.

In this study, we developed ^125^I-labeled DOX derivatives encapsulated in TSLs to achieve high accumulation in cancer tissues as liposomes, release of ^125^I-labeled DOX derivatives from TSLs by hyperthermia, and high accumulation of ^125^I-labeled DOX derivatives in the nuclei of cancer cells, causing cytotoxicity via Auger electrons emitted by ^125^I-labeled DOX derivatives (Figure 1). Based on cellular and nuclear uptake studies and cytotoxicity assays using Colon 26 cells, we demonstrated that high nuclear uptake was achieved when TSLs were used in combination with hyperthermia, leading to high cytotoxicity in cancer cells.

## 2. Results and Discussion

### 2.1. Synthesis of Non-Radioactive DOX Derivatives

**Scheme 1:**
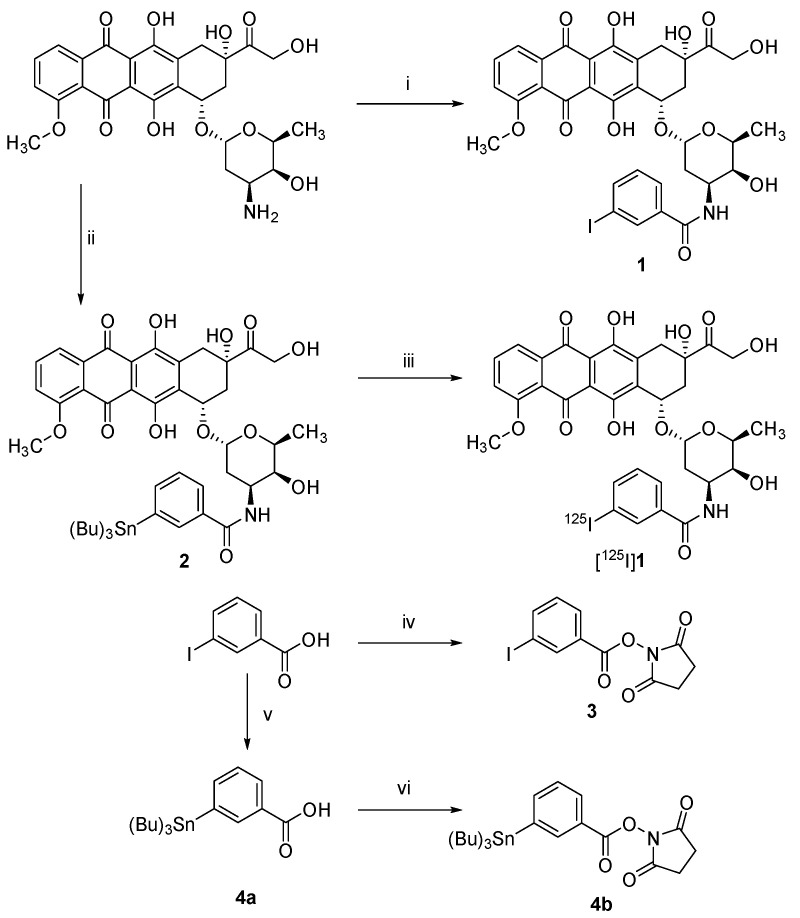
Synthetic scheme of **1** and [^125^I]**1**. Reagents and conditions: (i) **3**, triethylamine, DMF, rt; (ii) **4b**, triethylamine, DMF, rt; (iii) [^125^I]NaI, NCS, AcOH, NaHSO_3_, MeCN, water; (iv) 3-iodobenzoic acid, NHS, DCC, THF, rt; (v) dioxane, DSC, Pd[P(C_6_H_5_)_3_]_4_, 80 °C; and (vi) NHS, DCC, THF, rt.

Compounds **1** and **2** were synthesized as shown in Scheme 1. 4-Iodobenzoic acid and 4-(tributylstannyl)benzoic acid were reacted with di-(*N*-succinimidyl)carbonate in dry dioxane to yield the corresponding *N*-succinimidyl-derivatives (**7** and **8**, respectively), as described previously [19,20]. These activated compounds, **7** and **8**, were reacted with DOX hydrochloride in the presence of a fivefold molar excess of triethylamine in DMF to give 44% and 54% yields of **1** and **2**, respectively.

**Scheme 2:**
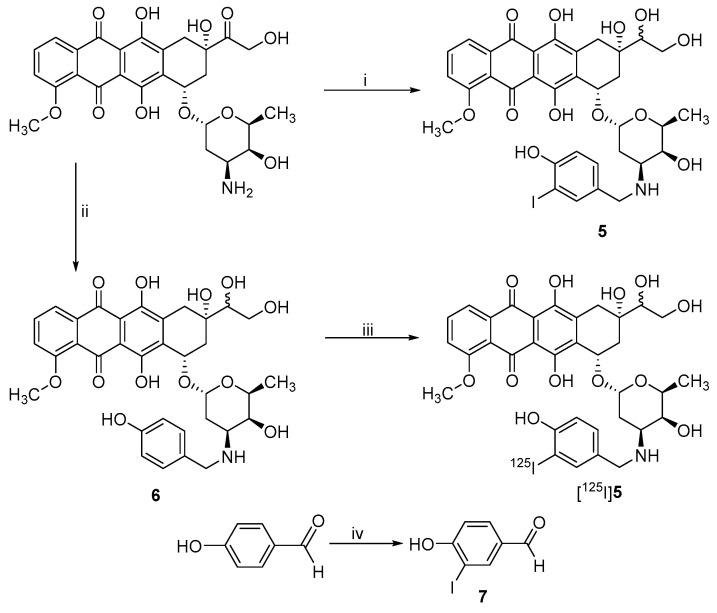
Synthetic scheme of **5** and [^125^I]**5**. Reagents and conditions: (i) Aldehyde **7**, MeCN-H_2_O (2:1), 1 M solution NaBH_3_CN/THF, rt; (ii) 4-hydroxybenzaoldhyde, MeCN-H_2_O (2:1), 1 M solution NaBH_3_CN/THF, rt; (iii) [^125^I]NaI, CAT, AcOH, NaHSO_3_, MeCN, water, 60 °C; and (iv) I_2_, Ag_2_SO_4_, CH_2_Cl_2_, rt.

Compounds **5** and **6** were synthesized as shown in Scheme 2. Compound **5** was prepared by the reductive amination of 4-hydroxybenzaldehyde with DOX hydrochloride using sodium cyanoborohydride to give a 44% yield of **5**. 4-Hydroxybenzaldehyde was iodinated using I_2_/Ag_2_SO_4_. The reaction afforded a moderate yield of the monoiodo derivative (**7**). Compound **6** was prepared from 4-hydroxy-3-iodobenzaldehyde (**7**) and DOX hydrochloride using a similar procedure to that used for the synthesis of **5** to give a 57% yield of **6.**

### 2.2. Intracellular Localization of DOX and DOX Derivatives

**Figure 2:**
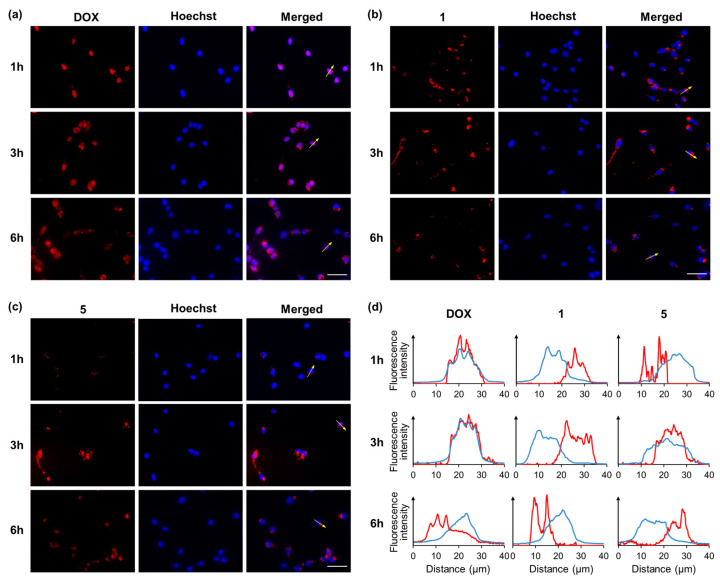
Intracellular localization of DOX derivatives. The fluorescent images were obtained at 1, 3, and 6 h after the addition of (**a**) DOX, (**b**) **1**, and (**c**) **5**. The fluorescence of DOX, **1**, and **5** is shown in red; the nucleus stained with Hoechst is shown in blue. (**d**) Line analysis of fluorescence signals of DOX-, **1**-, and **5**-treated cells (yellow arrow in merged images).

Intracellular localization of the DOX derivatives in Colon 26 cells was detected by fluorescence microscopy at 1, 3, and 6 h after the addition of DOX and DOX derivatives. The nuclei of Colon 26 cells were stained with Hoechst 33342. The accumulation of DOX derivatives in the nucleus was evaluated qualitatively by analyzing the colocalization of the fluorescence images of DOX derivatives with the nuclei (Hoechst 33342). To clarify the colocalization, line analyses were performed. Specifically, DOX accumulated in the nuclei of Colon 26 cells after 1 and 3 h (Figure 2a,d). However, almost no nuclear accumulation was observed for **1** (Figure 2b,d), whereas **5** partially accumulated in the nuclei after 3 h (Figure 2c,d). DOX has a primary amine structure that interacts with DNA [21]. To introduce ^125^I into DOX derivatives, the primary amine was converted to an amide (**1**) or secondary amine (**5**). Their decreased accumulation in the nucleus was probably due to the importance of the primary amine interacting with DNA. There also might be a steric hinderance by the introducing moiety for radiolabeling. Although the accumulation of **1** and **5** into the nuclei was not as high as that of DOX, the accumulation of **5** was greater than that of **1**.

### 2.3. Radiolabeling

[^125^I]**1** was synthesized by iododestannylation of the corresponding tributylstannyl precursor (**2**) using NCS as an oxidizing agent. The radiochemical yield was 78%. Radiolabeling of **6** was performed via direct electrophilic substitution with ^125^I using chloramine-T (CAT) as an oxidizing agent to give [^125^I]**5** with a radiochemical yield of 56%. After purification of [^125^I]**1** and [^125^I]**5** by reversed phased (RP)-HPLC, the radiochemical purity was >95%. These peaks were observed at the same retention times as those in the chromatograms (Figures S1 and S2).

### 2.4. Determination of the Partition Coefficient

The *n*-octanol/phosphate buffer (PB) partition coefficients as log *p*-values for [^125^I]**1** and [^125^I]**5** were 2.7 ± 0.1 and 1.9 ± 0.1, respectively, suggesting that [^125^I]**5** has a lower lipophilicity than [^125^I]**1**. Lipophilicity is typically utilized to predict and rationalize the in vivo behavior of probes, including membrane permeability and biodistribution. The membrane permeability of the probes is particularly important because the target molecule of the probes is situated in the intracellular domain. [^125^I]**1** and [^125^I]**5** possess appropriate lipophilicity for passive penetration of cellular membranes [22].

### 2.5. In Vitro Stability Assays

The stability of [^125^I]**1** and [^125^I]**5** in 0.1 M phosphate buffer saline (PBS) (pH 7.4) was evaluated by HPLC analysis, as shown in Figure S3. The radiochemical purities of [^125^I]**1** and [^125^I]**5** after 24 h incubation at 37 °C remained at 92.3 ± 0.2% and 93.2 ± 0.9%, respectively, revealing good probe stability in vitro.

### 2.6. Cellular and Nuclear Uptake of [^125^I]1 and [^125^I]5

**Figure 3:**
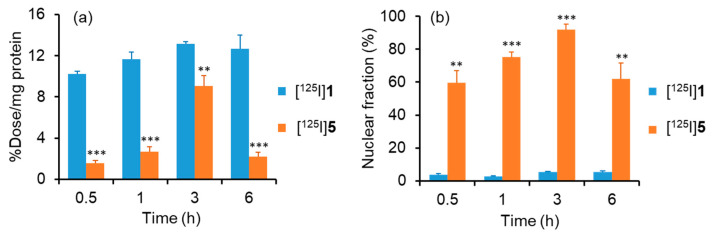
Cell uptake study using [^125^I]**1** and [^125^I]**5**. (**a**) The cellular uptake and (**b**) nuclear uptake ratio of [^125^I]**1** and [^125^I]**5** at 0.5, 1, 3, and 6 h. Data are presented as mean ± SD for three samples. ** *p* < 0.01, *** *p* < 0.001 vs. [^125^I]**1**.

In vitro cellular uptake experiments for [^125^I]**1** and [^125^I]**5** were performed using Colon 26 cells. As shown in Figure 3, the radioactivity uptake in the cells clearly increased over time during the first 3 h. Further increasing the incubation time to 6 h led to a slight decrease in the amount of radioactivity accumulated in the cells. The cellular uptake of [^125^I]**5** was lower than that of [^125^I]**1**, whereas the nuclear uptake ratio of [^125^I]**5** was significantly higher than that of [^125^I]**1**. The high cellular uptake of [^125^I]**1** was probably due to its high lipophilicity, as described above. The result of rather low uptake of [^125^I]**1** into the nuclei is consistent with the fluorescence images of **1** shown in Figure 2b. In the fluorescent images in Figure 2c, not so much, but rather partial accumulation of **5** into the nuclei was found, whereas 60–90% of [^125^I]**5** was detected in nuclear fractions. This discrepancy may have been caused by the rather different concentration between **5** and [^125^I]**5**. The concentration of [^125^I]**5** used for cellular and nuclear uptake studies was much lower than that of **5** used for fluorescence microscopy.

### 2.7. Auger Electron Therapy

**Figure 4:**
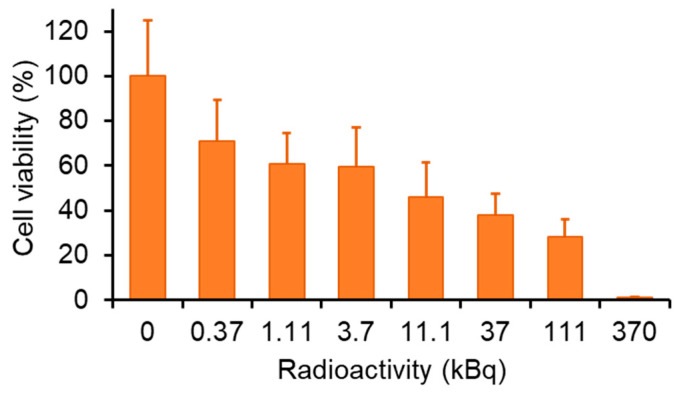
Cytotoxicity assay. The cytotoxicity of [^125^I]**5** toward Colon 26 cells. Data are presented as mean ± SD for 3–9 samples.

As shown in Figure 4, MTT assays were performed to evaluate the cytotoxicity of [^125^I]**5**. Cell viability decreased with the increasing radioactivity of [^125^I]**5**, and almost all cells died at 370 kBq. The cytotoxic effect of [^125^I]**5** was much more significant than that of non-radiolabeled compound **5.** The concentration of [^125^I]**5** at 370 kBq was 45.7 nM, calculated from the radioactivity and molar radioactivity of ^125^I. The IC_50_ value of **5** was 4.69 μM and no cytotoxic activity was observed at 45.7 nM, as shown in Figure S4. These results suggest that the cytotoxic effects of [^125^I]**5** were derived from Auger electron emitted from [^125^I]**5** in the vicinity of DNA.

### 2.8. Preperation of TSLs Encapsulating [^125^I]5

Liposomes composed of 1,2-dipalmitoyl-*sn*-glycero-3-phosphocholine (DPPC), Cholesterol (Chol), and 1,2-distearoyl-*sn*-glycero-3-phosphoethanolamine-*N*-[methoxy(polyethylene glycol)-2000] (ammonium salt) (PEG-DSPE) at molar ratios of 55:40:5 and 75:20:5, respectively, were prepared by the lipid film hydration method followed by extrusion. The phase transition temperature of liposomes was analyzed by differential scanning calorimetry. The phase transition temperature of the liposome composed of DPPC, Chol, and PEG-DSPE at a molar ratio of 75:20:5 was 43.6 °C, whereas the phase transition peak was not observed for the liposome composed of DPPC, Chol, and PEG-DSPE at a molar ratio of 55:40:5. From these results, we treated them as TSLs and non-thermosensitive liposomes (NTLs), respectively. The onset of the phase transition was observed at 38.1 °C for TSLs.

NTLs and TSLs were loaded with [^125^I]**5** using the pH gradient-driven loading protocol. High encapsulation efficiencies of [^125^I]**5** in NTLs (78%) and TSLs (75%) were found. The loading efficiencies of [^125^I]**5** in NTLs and TSLs were 3.9% and 4.2%, respectively.

### 2.9. Drug Release Test

**Figure 5:**
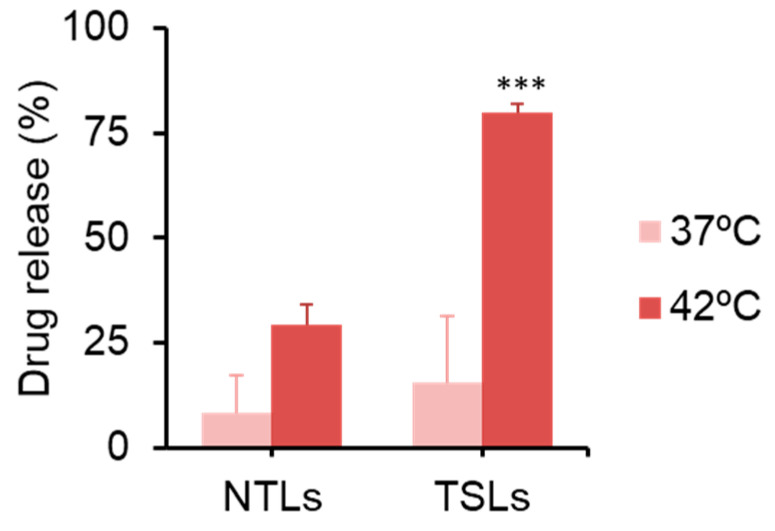
Drug release test. The release of [^125^I]**5** from NTLs and TSLs was analyzed in 90% FBS at 37 °C for 1 h or 42 °C for 10 min. Data are presented as mean ± SD for three samples. *** *p* < 0.001 vs. other conditions.

The release of [^125^I]**5** from NTLs and TSLs was studied in 90% fetal bovine serum (FBS) at 37 °C for 1 h or 42 °C for 10 min. [^125^I]**5** showed rather low drug release from NTLs (8.2%) and TSLs (15.6%) at 37 °C. Although the drug release from NTLs at 42 °C (29%) was relatively high compared with the release at 37 °C, 80% of [^125^I]**5** was released from TSLs when incubated at 42 °C (Figure 5). These results suggest that TSLs encapsulating [^125^I]**5** show relatively high stability at body temperature and effectively release [^125^I]**5** when used in combination with hyperthermia.

### 2.10. Cellular and Nuclear Uptake of TSLs Encapsulating [^125^I]5

**Figure 6:**
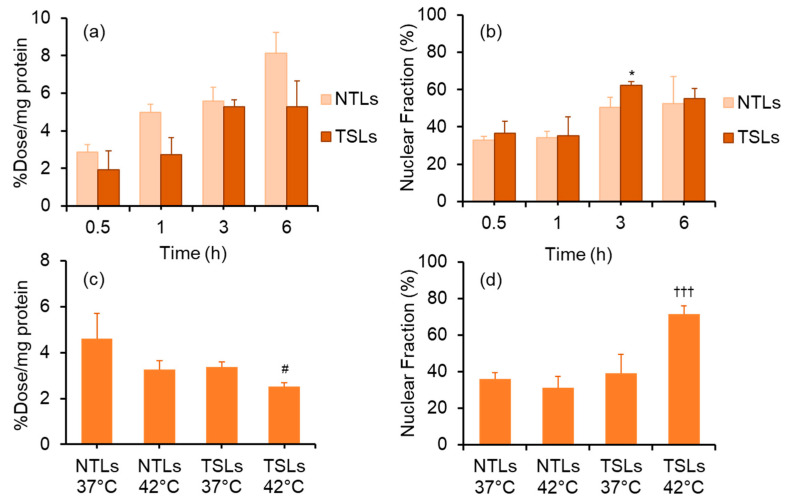
Cell uptake study using TSLs encapsulating [^125^I]**5**. (**a**) The cellular uptake of NTLs and TSLs encapsulating [^125^I]**5** and (**b**) nuclear uptake ratio of NTLs and TSLs encapsulating [^125^I]**5** at 37 °C at 0.5, 1, 3, and 6 h. (**c**) The cellular uptake and (**d**) nuclear uptake ratio of NTLs and TSLs encapsulating [^125^I]**5** at 37 or 42 °C for 1 h. Data are presented as mean ± SD for three samples. * *p* < 0.05 vs. NTLs, ^#^ *p* < 0.05 vs. NTLs at 37 °C, ^†††^ *p* < 0.001 vs. other conditions.

To evaluate the delivery of [^125^I]**5** released from TSLs into the nucleus, in vitro cellular and nuclear uptake experiments were performed using the Colon 26 cell line. As shown in Figure 6, radioactivity uptake in cells gradually increased over time for both NTLs and TSLs encapsulating [^125^I]**5**, and the radioactivity uptake ratio into the nuclei of the cells treated with NTLs and TSLs encapsulating [^125^I]**5** was about 30–60%. The nuclear uptake ratios were lower than those for [^125^I]**5**, which was not encapsulated in the liposomes (Figure 3). To evaluate whether [^125^I]**5** was released from TSLs with hyperthermia and whether it accumulated in the nuclei of cancer cells, the cellular and nuclear uptake of radioactivity was determined 1 h after adding NTLs or TSLs with or without hyperthermia. The cellular uptake of radioactivity was not so different among the studied conditions, although a significant difference was observed between NTLs at 37 °C and TSLs at 42 °C. However, the nuclear uptake ratio for TSLs at 42 °C was much higher than in other conditions, suggesting that the TSLs encapsulating [^125^I]**5** in combination with hyperthermia would effectively deliver the ^125^I to the nuclei in cancer cells.

## 3. Materials and Methods

### 3.1. Materials

DOX was purchased from LC Laboratories (Woburn, MA, USA). 1,2-Dipalmitoyl-*sn*-glycero-3-phosphocholine (DPPC) and 1,2-distearoyl-*sn*-glycero-3-phosphoethanolamine-*N*-[methoxy(polyethylene glycol)-2000] (ammonium salt) (PEG-DSPE) were purchased from NOF Corporation (Tokyo, Japan). Cholesterol (Chol) was purchased from Sigma Aldrich (St. Louis, MO, USA). [^125^I]Sodium iodide (644 GBq/mg) was purchased from PerkinElmer (Waltham, MA, USA). Other reagents were of reagent grade and used as received. Thin layer chromatography (TLC) was performed on silica plates 60 F254 (Merck, Darmstadt, Germany). Nuclear magnetic resonance (NMR) spectra were recorded on a JNM ECS400 (400 MHz) or JNM ECA600 (600 MHz) spectrometer (JEOL Ltd., Tokyo, Japan). The mass spectrometry was performed on a JMS T700 (JEOL Ltd.,) on electrospray ionization mass spectrometry (ESI-MS). HPLC analyses and purification were carried out on a Shimadzu Prominence HPLC system (Shimadzu Corp., Kyoto, Japan). Radioactivity was determined using an auto gamma counter ARC 7010B (Hitachi, Ltd., Tokyo, Japan). A colorectal adenocarcinoma cell line, Colon 26, was obtained from Cell Resource Centre for Biomedical Research in Tohoku University.

### 3.2. Synthesis of Non-Radioactive Compounds

#### 3.2.1. Synthesis of 3-(tri-n-butylstannyl)benzoic Acid (**4a**)

To a stirred solution of 3-iodobenzoic acid (102 mg, 0.41 mmol) in dry dioxane (10 mL), di-(*N*-succinimidyl)carbonate (0.48 mL, 0.964 mmol) and tetrakis(triphenylphosphine)palladium (Pd[P(C_6_H_5_)_3_]_4_) (4 mg, 0.04 mmol) were added under a nitrogen atmosphere at 80 °C, and the mixture was stirred for 24 h. After completion of the reaction, solvent was removed under reduced pressure and purified by flash column chromatography on silica gel (hexane/ethyl acetate = 3/1) to obtain **4a** (94.5 mg, 57%) as a colorless oil.

#### 3.2.2. Synthesis of *N*-Succinimidyl 3-(tri-n-butylstannyl)benzoate (ATE) (**4b**)

A mixture of **8a** (90 mg, 0.21 mmol), *N*-hydroxysuccinimide (NHS) (32.5 mg, 0.28 mmol), and *N*,*N*’-dicyclohexylcarbodiimide (DCC) (57.7 mg, 0.28 mmol) in dry THF (3 mL) was stirred at room temperature overnight. After removing the formed precipitate by filtration. The filtrate was concentrated under reduced pressure and purified by flash column chromatography on silica gel (hexane/ethyl acetate = 3/1) to obtain **4b** (41 mg, 38%) as a colorless oil. ^1^H NMR (400 MHz, DMSO-d_6_): δ 8.13 (1H, t, *J* = 1.6 Hz), 8.03 (1H, dd, *J* = 7.6, 1.6 Hz), 7.91 (1H, dd, *J* = 7.6, 1.6 Hz), 7.62 (1H, t, *J* = 7.6 Hz), 2.90 (4H, s), 1.42–1.62 (6H, m), 1.24–1.34 (6H, m), 0.98–1.13 (6H, m), 0.83–0.90 (9H, m).

#### 3.2.3. Synthesis of *N*-succinimidyl 3-iodobenzoate (SIB) (**3**)

To a solution of 3-iodobenzoic acid (100 mg, 0.4 mmol) in dry THF (4 mL) in an ice bath, NHS (58 mg, 0.5 mmol) was added and cooled for 5 min. To the mixture, DCC (103 mg, 0.5 mmol) was added, and the mixture was stirred at room temperature overnight. After removing the formed precipitate by filtration, the filtrate was concentrated under reduced pressure and purified by flash column chromatography on silica gel (hexane/ethyl acetate = 3/1) to obtain **3** (110 mg, 80%) as a white solid. ^1^H NMR (400 MHz, CDCl_3_): δ 8.47 (1H, s), 8.11 (1H, d, *J* = 7.6 Hz), 8.02 (1H, d, *J* = 7.6 Hz), 7.27 (1H, t, *J* = 7.6 Hz), 2.92 (4H, s).

#### 3.2.4. Synthesis of 3’-*N*-3-Iodo-benzoyldoxorubicin (**1**)

To a solution of doxorubicin hydrochloride (5 mg, 8.6 µmol) in dry *N,N*-dimethylformamide (DMF) (600 µL), SIB (3 mg, 8.6 µmol) in dry DMF (500 µL) was added under a nitrogen atmosphere. A sevenfold molar excess of triethylamine (6 mg, 60.2 µmol) was added to the reaction mixture and the mixture was stirred at room temperature under a nitrogen atmosphere for 24 h. After completion of the reaction, the mixture was concentrated under reduced pressure and purified by flash column chromatography on silica gel (ethyl acetate/pentane = 7/3) to obtain **1** (2.9 mg, 44%) as a red solid. ^1^H NMR (400 MHz, CDCl_3_): δ 8.05 (1H, d, *J* = 8.0 Hz), 7.80 (1H, t, *J* = 8.4 Hz), 7.72–7.67 (2H, m), 7.52 (1H, q, *J* = 7.6 Hz), 7.46 (1H, dd, *J* = 2.4 Hz), 7.40 (1H, dd, *J* = 4.0, 2.4 Hz), 7.15 (1H, t, *J* = 12.0 Hz), 6.38 (1H, d, *J* = 8.4 Hz), 5.55 (1H, d, *J* = 4.4 Hz), 5.33 (1H, s), 4.77 (1H, d, *J* = 4.4 Hz), 4.55 (1H, s), 4.34 (1H, s), 4.21 (2H, q, *J* = 6.4 Hz), 4.08 (1H, s), 3.74 (1H, s), 3.65 (2H, d, *J* = 8 Hz), 3.49 (1H, s), 3.07–3.05 (2H, m), 2.34 (1H, d, *J* = 16 Hz), 2.22 (1H, dd, *J* = 4 Hz), 2.02–1.98 (3H, m), 1.90–1.84 (1H, m). LRMS (ESI^+^): *m/z* calcd for [M+Na]^+^: *m/z* = C_34_H_32_INO_12_Na, 796.1, found, 796.1.

#### 3.2.5. Synthesis of 3’-*N*-3-Tributylstannylbenzoyldoxorubicin (**2**)

The synthesis of **2** was carried out as described for **1** above, except that *N*-succinimidyl-4-(tributylstannyl)benzoic acid (ATE) was used as the starting material. The reaction was completed after stirring at room temperature overnight. The reaction mixture was concentrated under reduced pressure and purified by flash column chromatography on silica gel (dichloromethane/methanol = 10/1) to obtain **2** (4.2 mg, 54%) as a red solid. ^1^H NMR (400 MHz, CDCl_3_):δ 8.02 (1H, t, *J* = 8.0 Hz), 7.81–7.77 (3H, m), 7.61–7.55 (2H, m), 7.38 (1H, dd, *J* = 7.2, 4.0 Hz), 7.33 (1H, dd, *J* = 4.0 Hz), 6.44 (1H, d, *J* = 8.0 Hz), 5.55 (1H, d, *J* = 4.0 Hz), 5.32 (1H, s), 4.85–4.78 (1H, m), 4.61 (1H, s), 4.40–4.25 (3H, m), 4.08 (2H, s), 3.74 (1H, s), 3.71 (1H, t, *J* = 16.0 Hz), 3.63 (1H, q, *J* = 4.0 Hz), 3.32 (1H, d, *J* = 16.9 Hz), 3.05 (1H, t, *J* = 12.6 Hz), 2.36 (2H, t, *J* = 14.4 Hz), 2.21 (1H, q, *J* = 2.4 Hz), 2.01 (1H, q, *J* = 2.0 Hz), 1.86 (1H, q, *J* = 12.8 Hz), 1.53–1.45 (3H, m), 1.35–1.25 (12H, m), 1.07–1.03 (6H, m), 0.90–0.84 (9H, m). LRMS (ESI^+^): *m/z* calcd for [M+Na]^+^: *m/z* = C_46_H_59_NO_12_SnNa, 960.3, found, 960.4.

#### 3.2.6. 4-Hydroxy-3-iodobenzaldehyde (**7**)

To a solution of 4-hydroxybenzaldehyde (50 mg, 0.41 mmol) in dichloromethane, Ag_2_SO_4_ (166 mg, 0.53 mmol) and I_2_ (134 mg, 0.51 mmol) were added and stirred at room temperature overnight. After removing the formed precipitate by filtration, the filtrate was concentrated under reduced pressure and purified by flash column chromatography on silica gel (hexane/ethyl acetate = 3/1) to obtain **7** (23.5 mg, 24%) as a colorless solid. ^1^H NMR (400 MHz, DMSO-d_6_): (400 MHz, CDCl_3_): δ 9.73 (1H, d, *J* = 1.2 Hz), 8.22 (1H, s), 7.75 (1H, t, *J* = 8 Hz), 7.09 (1H, d, *J* = 8.6 Hz).

#### 3.2.7. 3’-*N*-(4-Hydroxy-3-iodobenzyl)-13-(R/S)-dihydrodoxorubicin (**5**)

To a solution of DOX hydrochloride (8 mg, 13.8 µmol) and 4-hydroxy-3-iodobenzaldehyde (20.1 mg, 82.8 µmol) in a 2:1 ratio mixture of acetonitrile and water (3 mL), 1M THF solution of sodium cyanoborohydride (55.2 µL, 55.2 µmol) was added. The reaction mixture was stirred at room temperature in the dark under a nitrogen atmosphere for 48 h. After completion of the reaction, the mixture was concentrated under reduced pressure and purified by flash chromatography on silica gel (dichloromethane/methanol = 7/1) to obtain **5** (3.6 mg, 57%) as a red solid. ^1^H NMR (400 MHz, CDCl_3_): δ 7.47 (1H, s), 7.31 (1H, s), 7.22 (1H, m), 6.87 (1H, d, *J* = 12.0 Hz), 6.64 (2H, s), 6.22 (2H, s), 5.01 (2H, s), 4.64 (2H, s), 3.44 (1H, d, *J* = 8.8 Hz), 3.28 (2H, t, *J* = 12.4 Hz), 3.22 (2H, q, *J* = 8.4 Hz), 3.10–3.02 (1H, m), 2.63–2.59 (1H, m), 2.36 (3H, s), 1.71 (1H, t, *J* = 12.8 Hz), 1.43 (3H, d, *J* = 5.2 Hz). LRMS (ESI^+^): *m/z* calcd for [M+Na]^+^: *m/z* = C_34_H_36_INO_11_Na, 800.1, found, 800.0.

#### 3.2.8. 3’-*N*-(4-Hydroxybenzyl) -13-(R/S)-dihydrodoxorubicin (**6**)

To a solution of doxorubicin hydrochloride (8 mg, 13.8 µmol) and 4-hydroxybenzaldehyde (10.12 mg, 82.8 µmol) in a 2:1 ratio mixture of acetonitrile and water (3 mL), 1M THF solution of sodium cyanoborohydride (55.2 µL, 55.2 µmol) was added. The reaction mixture was stirred at room temperature in the dark under a nitrogen atmosphere for 48 h. After completion of the reaction, the mixture was concentrated under reduced pressure and purified by flash chromatography on silica gel (dichloromethane/methanol = 7/1) to obtain **6** (4 mg, 71%) as a red solid. ^1^H NMR (400 MHz, CDCl_3_): δ 7.47 (1H, s), 7.32–7.28 (2H, m), 6.87 (1H, d, *J* = 12.0 Hz), 6.63 (2H, s), 6.25 (2H, s), 5.01 (2H, s), 4.58 (2H, s), 3.44 (1H, d, *J* = 8.8 Hz), 3.28 (2H, t, *J* = 12.4 Hz), 3.22 (2H, q, *J* = 8.4 Hz), 3.10–3.01 (1H, m), 2.73–2.67 (1H, m), 2.36 (3H, s), 1.71 (1H, t, *J* = 12.8 Hz), 1.43 (3H, d, *J* = 5.2 Hz). LRMS (ESI^+^): *m/z* calcd for [M+H]^+^: *m/z* = C_34_H_35_NO_12_: 652.2, found, 652.3.

### 3.3. Synthesis of Radioactive Compounds

#### 3.3.1. Radiosynthesis of 3’-*N*-3-iodo-benzoyldoxorubicin

A solution of [^125^I]NaI (370 kBq, 2 µL) was added into a sealed vial containing tributylstannyl precursor (**2**) (80 µg), acetic acid (25%) in acetonitrile (70 µL), and NCS (10 mg/mL, 20 µL). The mixture was shaken at room temperature for 5 min and quenched by the addition of sodium hydrogensulfite (8 mg/mL, 10 µL) and 10 μL of NaI (10 mg/mL solution in H_2_O) and analyzed by HPLC.

#### 3.3.2. Radiosynthesis of 3’-*N*-(4-hydroxy-3-iodobenzyl)doxorubicin

A solution of [^125^I]NaI (370 kBq, 2 µL) was added into a sealed vial containing precursor (**6**) (80 µg), acetic acid (25%) in acetonitrile (50 µL), and CAT (20 mg/mL, 15 µL). The mixture was shaken at 60 °C for 5 min, quenched by the addition of sodium hydrogensulfite (8 mg/mL, 10 µL) and 10 μL of NaI (10 mg/mL solution in H_2_O), and analyzed by HPLC.

### 3.4. Intracellular Localization

Colon 26 cells were cultured in RPMI-1640 medium supplemented with 10% heat-inactivated fetal bovine serum (FBS), penicillin (100 units/mL), and streptomycin (100 mg/mL) at 37 °C in a 5% CO_2_ humidified atmosphere. Colon 26 cells were seeded into eight-well glass chamber at a density of 3000 cells/well and incubated overnight. Cells were treated with the medium containing 3 µM DOX and DOX derivatives for 1, 3, and 6 h and at 37 °C in a humidified 5% CO_2_ atmosphere. After washing twice with 0.2 mL of cold PBS, 0.2 mL of Hoechst33342 solution (2.5 μg/mL) was added and incubated for 10 min. After rinsing with PBS, fluorescence microscopy was carried out using BZ-X800 All-in-One Fluorescence Microscope (KEYENCE, Osaka, Japan) to detect the intracellular localization of DOX and DOX derivatives (excitation λ = 360/40 nm and emission λ = 460/50 nm for nuclei; excitation λ = 545/25 nm and emission λ = 605/70 nm for DOX and DOX derivatives). Using the ImageJ v1.53e software (National Institutes of Health), quantitative line analysis was performed.

### 3.5. Determination of Partition Coefficients

Partition coefficients of [^125^I]**1** and [^125^I]**5** into *n*-octanol and 0.1 M PB pH 7.4 were determined as previously described with a slight modification [23]. In brief, [^125^I]**1** or [^125^I]**5** was added to the combination of *n*-octanol (3.0 mL) and PB (3.0 mL) in a test tube. The test tube was vortexed (1 min), remaining at room temperature (15 min), and centrifuged (5 min, 1000× *g*, 4 °C). The *n*-octanol layer (2.5 mL) was moved into a brand new test tube accompanied by the addition of fresh *n*-octanol (0.5 mL) and PB (3.0 mL). After repeated vortex, centrifuge, and standing, the radioactivity of each layer, *n*-octanol (2.0 mL) and PB (2.0 mL), was measured using an auto-well gamma counter (*n* = 4).

### 3.6. In Vitro Stability Assay

The stability of radiotracers, [^125^I]**1** and [^125^I]**5**, were examined according to the previously reported method with a minor modification [24]. Briefly, [^125^I]**1** or [^125^I]**5** solution (10 μL) in Eppendorf tube containing 0.1 M phosphate buffered saline (PBS) pH 7.4 (90 μL) was incubated at 37 °C for 1 and 24 h. The purities of radiotracers after incubation were determined by HPLC.

### 3.7. Cytotoxicity Assay

The cytotoxicity of **1**, **5**, [^125^I]**1**, and [^125^I]**5** to Colon 26 cells was examined utilizing the 3-(4,5-dimethylthiazol-2-yl)-2,5-diphenyltetrazolium bromide (MTT)-based cytotoxicity assay. Briefly, cells were seeded into 96-well plates at a density of 3000 cells/well. The plates were incubated for 24 h at 37 °C in a humidified 5% CO_2_ atmosphere. Nonradioactive and radioactive DOX derivatives (**1** and **5**: 0, 0.001, 0.01, 0.1, 0.3, 1, 3, 10, 33, 100 µM, [^125^I]**1** and [^125^I]**5**: 0, 0.37, 1.11, 3.7, 11.1, 37, 111, 370 kBq) were placed into each corresponding well in the plates. After incubation for 48 h, every well was flushed and rinsed with growth medium. MTT solution (5 mg/mL) was added into each well and the plates were incubated for 4 h at 37 °C. The medium containing MTT was taken out of the wells and the remaining MTT formazan crystals were dissolved with the addition of 100 μL of DMSO. After that, each plate was set into a microplate reader and absorbance at 570 nm was measured.

The IC_50_ value was defined as the drug concentration that reduced the relative absorbance by 50% of drug-free control. Relative absorbance was calculated according to the following equation: RA = (SA − BA)/(CA − BA), where RA is the relative absorbance, SA is the observed absorbance of the sample, CA is the observed absorbance of the control (drug-free), and BA is the observed absorbance of the blank (drug- and cell-free, only medium).

### 3.8. Preparation of Liposomes

Liposomes composed of DPPC, Chol, and PEG-DSPE at a molar ratio of 55:40:5 for NTLs and 75:20:5 for TSLs were prepared by the lipid film hydration method, as described previously [25]. Chol and phospholipids were dissolved in chloroform in a round bottom flask, followed by the evaporation using a rotary evaporator, and the lipid film was dried under a vacuum for 1 h. The lipid film was hydrated with 300 mM citrate buffer (pH 4) with vortex before extrusion. Then, the resulting multilamellar preparation was sized by repeated extrusion through polycarbonate membrane filters (Whatman Inc. Nucleopore, Newton, MA) with a pore size of 100 nm using an Avanti Mini-extruder (Avanti Polar Lipids Inc., Alabaster, AL, USA). The resulting liposomes were passed through a Sephadex G-25 column (PD-10 desalting column, GE Healthcare, Little Chalfont, UK) equilibrated with PBS (pH 7.4) to change the pH of the external phase. The lipid concentration was determined using the phospholipid determination kit.

### 3.9. Differential Scanning Calorimetry

The thermal behavior of the liposomes was investigated using a DSC 250 (TA Instruments, New Castle, DE, USA). Liposomes were prepared in a concentration of 24 mM and 20 μL transferred in a Tzero aluminium pan (TA Instruments) and hermetically sealed. The applied heating rate was 5 °C/min. After a first heating above the phase transition temperature, two additional cycles were performed. The last cycle was used for the evaluation of the thermal profile and the phase transition temperature.

### 3.10. Drug Encapsulation

The liposomes were loaded with [^125^I]**5** using the pH-gradient-driven loading protocol described by Mayer et al. [26]. The pH gradient across the liposome membrane was generated by exchanging the extravesicular 300 mM citrate buffer (pH 4) with PBS (pH 7.4) using a PD-10 column. Preheated drug solution was added to liposomes at a 0.05 drug-to-lipid molar ratio. The mixtures were incubated for 60 min at 37 °C. Unencapsulated drug was removed by passing through a PD-10 column equilibrated with PBS (pH 7.4). The content in the liposome fractions was determined by gamma counting. The encapsulation efficiency of [^125^I]**5** in liposomes (%) is the radioactivity of encapsulated [^125^I]**5**/radioactivity of [^125^I]**5** initially added × 100, while the loading efficiency of [^125^I]**5** in liposomes (%) is the weight of encapsulated [^125^I]**5**/weight of liposomes × 100.

### 3.11. Drug Release Test

The release of [^125^I]**5** from NTLs and TSLs was studied in 90% FBS at a temperature of 37 or 42 °C. Liposomes with a total lipid concentration of 1 mmol/L were used. Samples were taken after the addition of liposomes to the FBS for 1 h at 37 °C and 10 min at 42 °C. Liposomes were separated from the unencapsulated drug on PD-10 columns and the [^125^I]**5** content in the liposome fractions was determined by gamma counting.

### 3.12. Cellular and Nuclear Uptake Study

Colon 26 cells was seeded on six-well plates at a density of 1 × 10^6^ cells/well for 24 h. After the removal of medium, a solution of [^125^I]**1** or [^125^I]**5** (3.7 kBq/well) in medium was added and incubated for 0.5, 1, 3, and 6 h at 37 °C, while for liposome encapsulating [^125^I]**5** (0.25 µmol liposomes loaded with [^125^I]**5**), it was incubated for 1 h at 37 °C or 42 °C. After incubation, the medium was eliminated and the cells were washed once with ice cold PBS (1 mL). The cells were collected using trypsin (1 mL). The radioactivity of the cellular pellet was measured with a gamma counter to evaluate the overall cellular uptake of the radio compound. The pellet was then resuspended in 2 mL of ice-cold cell lysis buffer (10 mM Tris, 1.5 mM MgCl_2_, 140 mM NaCl, and 0.01% Triton X 100) and incubated for 10 min on an ice bath to disrupt the cell membrane. Following lysis, the suspension was centrifuged at 4 °C for 2 min at 1300× *g*. The supernatant (cytoplasm) was separated from the pellets (nuclei) and the activity was measured in both fractions. Nuclear uptake was expressed in internalized activity [27]. The total cell protein was quantified using a BCA Protein Assay Kit (Nacalai Tesuque, Kyoto, Japan) following the manufacturer’s protocol and bovine serum albumin was used as a protein standard. All data were expressed as percent dose per milligram protein (% dose/mg protein).

### 3.13. Statistical Analysis

All data were examined utilizing Graph Pad Prism 5.0 (GraphPad Software, San Diego, CA, USA). Unpaired Student’s *t*-test was utilized to confirm the significances within the cytotoxicity assays and the cellular uptake studies. One-way ANOVA with Tukey’s multiple comparison test was performed for the drug release test and the cellular uptake studies. Most values are provided as mean ± SD and *p* < 0.05 was considered statistically significant.

## 4. Conclusions

In this study, we developed a thermosensitive liposome encapsulating ^125^I-labeled DOX derivative [^125^I]**5** for Auger electron therapy. We synthesized ^125^I-labeled DOX derivatives [^125^I]**1** and [^125^I]**5** with high radiochemical purities (no less than 95%). The cellular uptake of [^125^I]**5** in Colon 26 cells was lower than that of [^125^I]**1**, whereas the nuclear uptake ratio of [^125^I]**5** was much higher than that of [^125^I]**1**. The high accumulation into the nuclei of cancer cells resulted in high cytotoxicity of [^125^I]**5** owing to the Auger electron emitted from ^125^I. From these results, [^125^I]**5** was loaded in TSLs with high encapsulation efficiencies. High drug release was achieved for TSLs encapsulating [^125^I]**5** in combination with hyperthermia. The uptake ratio of radioactivity into the nuclei of Colon 26 cells treated with TSLs encapsulating [^125^I]**5** was lower than that of the cells treated with [^125^I]**5**. However, a high uptake ratio in the nuclei of the cells treated with TSLs encapsulating [^125^I]**5** was found when heating at 42 °C for 1 h. The high nuclear uptake ratio suggested that [^125^I]**5** was released by heating at 42 °C and accumulated in the nuclei of the cells. These results suggested that the combination of TSLs encapsulating [^125^I]**5** with hyperthermia would deliver ^125^I to the nuclei and kill cancer cells.

## Data Availability

Not applicable.

## Supplementary Materials

The following supporting information can be downloaded at https://www.mdpi.com/article/10.3390/molecules28041864/s1. Figure S1: RP-HPLC chromatograms of (a) nonradioactive iodinated compound 1 and (b) radioactive compound [^125^I]**1**. Figure S2: RP-HPLC chromatograms of (a) nonradioactive iodinated compound **5** and (b) radioactive compound [^125^I]**5**. Figure S3: The stability of [^125^I]**1** and [^125^I]**5** in PBS. Figure S4: Cytotoxicity assay. The cytotoxicity of **5** toward Colon 26 cells.

## Sample Availability

Samples of the compounds are not available from the authors.
