# Supplementary material for: Preparation and Evaluation of Thermosensitive Liposomes Encapsulating I-125-Labeled Doxorubicin Derivatives for Auger Electron Therapy
Source: Molecules. 2023 Feb 16;28(4):1864. doi: 10.3390/molecules28041864 (PMC9962004; doi:10.3390/molecules28041864)
Supplement: Supplementary file 1 [file molecules-28-01864-s001.zip › molecules-2186149-supplementary.pdf]

Supporting Information

Preparation and Evaluation of Thermosensitive Liposomes Encapsulating I-125-Labeled Doxorubicin Derivatives for Auger Electron Therapy

Mohamed Elsaid Nasr Elghobary [1,†], Masayuki Munekane [1,*,†], Kenji Mishiro [2], Takeshi Fuchigami [1] and Kazuma Ogawa [1,2,*]

[1] Graduate School of Medical Sciences, Kanazawa University, Kanazawa 920-1192, Japan
[2] Institute for Frontier Science Initiative, Kanazawa University, Kanazawa 920-1192, Japan
* Correspondence: munekane@p.kanazawa-u.ac.jp (M.M.); kogawa@p.kanazawa-u.ac.jp (K.O.); Tel./Fax: +81-76-234-4461 (M.M.); +81-76-234-4460 (K.O.)
† These two authors contributed equally to this work.

Table of Contents

1. HPLC analysis……………………………………………………………….. S1-2

2. Stability test …………………………………………..………………… S3

3. MTT assay ……………………………………………..……………… S4

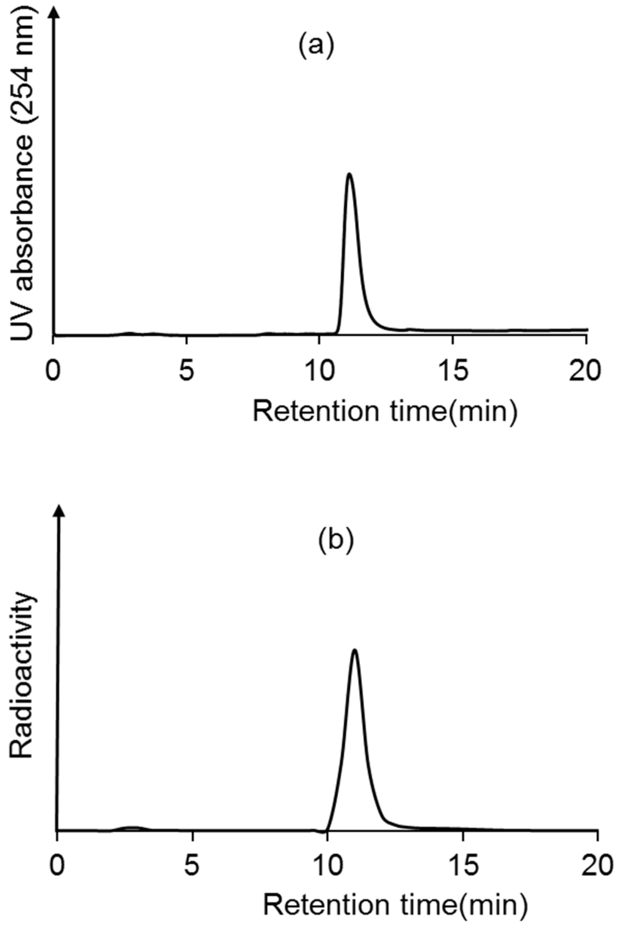

Figure S1. RP-HPLC chromatograms of (a) nonradioactive iodinated compound **1** and (b) radioactive compound [^{125}I]**1**.

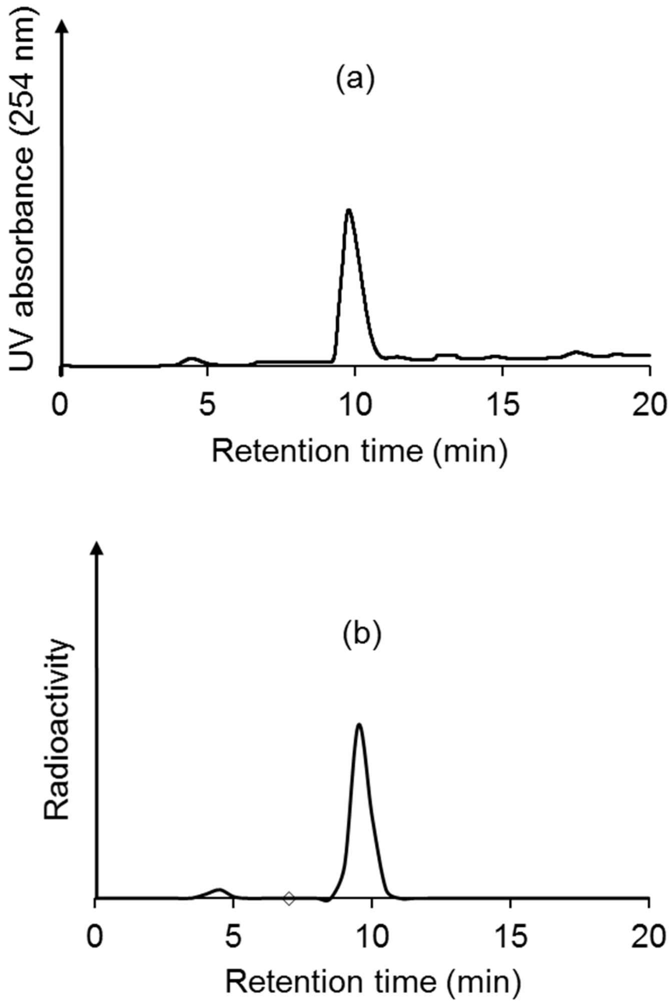

Figure S2. RP-HPLC chromatograms of (a) nonradioactive iodinated compound **5** and (b) radioactive compound [^{125}I]**5**.

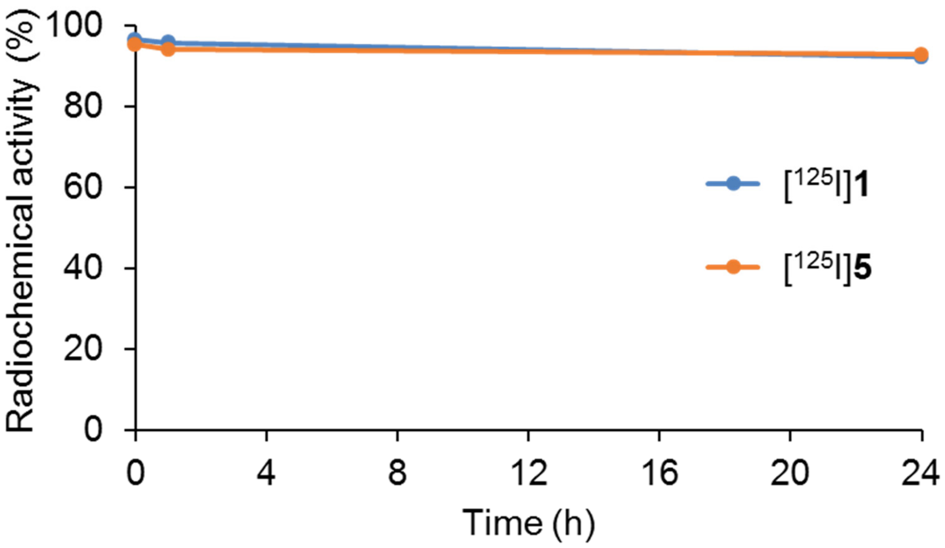

Figure S3. The stability of [^{125}I]**1** and [^{125}I]**5** in PBS.

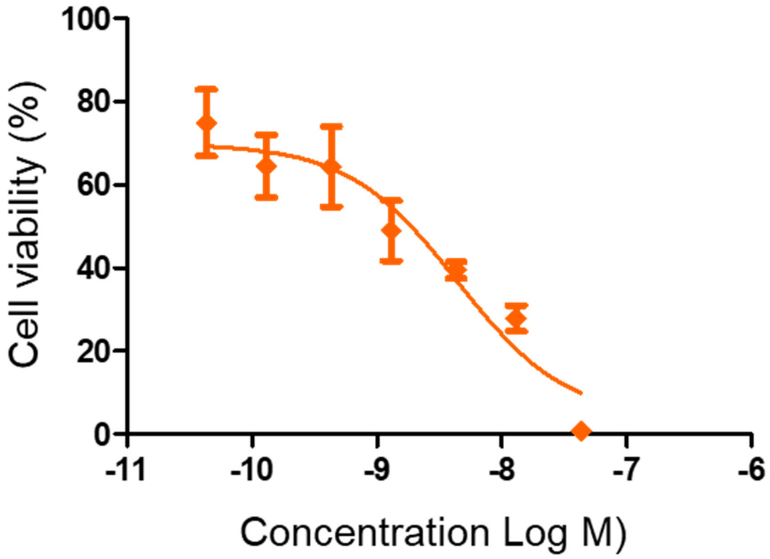

Figure S4. Cytotoxicity assay. The cytotoxicity of **5** toward Colon 26 cells. Data are presented as mean ± SD for 3 samples.
